# The causal relationship between mental illness-related stigma and mental health knowledge among adolescents at different educational stages: a longitudinal cross-lagged study

**DOI:** 10.3389/fpsyt.2025.1726878

**Published:** 2026-01-14

**Authors:** Yi-Yue Yang, Ke Zhao, Cong Wang, Yi-Hao Liu, Lie Zhou, Hui Jin, Yun Xiao, Yang Wen, Jawad Ahmad, Yun-Fei Mu, Michelle Yu-Xin Ran, Zi-Qi Wang, Xiao-Fei Zhou, Jia Cai, Bo Tian, Mao-Sheng Ran

**Affiliations:** 1 Mental Health Center, West China Hospital, Sichuan University, Chengdu, Sichuan, China; 2 Department of Social Psychiatry, West China Hospital, Sichuan University, Chengdu, Sichuan, China; 3 Chongqing Mental Health Center, Chongqing, China; 4 Chengdu Fourth People’s Hospital, Chengdu, Sichuan, China

**Keywords:** adolescent, causal relationship, different educational stages, mental health knowledge, mental illness-related stigma

## Abstract

**Background:**

The relationship between mental illness knowledge and mental illness-related stigma among adolescent and youth students remains unclear. This study aims to examine the longitudinal predictive relationship between mental illness knowledge and mental illness-related stigma among junior high school, high school, and university students in China.

**Methods:**

A longitudinal study design was employed. Two questionnaire surveys were conducted in December 2022 (T1) and December 2023 (T2) among 5,579 adolescent and youth students (1,695 junior high, 3,120 high school, and 764 university students) in Sichuan Province. The Mental Health Knowledge Schedule (MAKS) and the Perceived Devaluation-Discrimination Scale (PDD) were used. Cross-lagged panel models were applied to analyze the mutual predictive relationships between the variables.

**Results:**

At baseline, significant differences were observed in demographic characteristics and scores on the MAKS and PDD among the three group students (p < 0.05). Cross-lagged model analysis revealed that across all three educational stages, the scores of MAKS at T1 significantly positively predicted the scores of PDD at T2 (p < 0.05), and significantly negatively predicted MAKS at T2 (p < 0.05). This predictive pattern was the most pronounced in the university student group.

**Conclusion:**

An increase in mental health knowledge may not alleviate mental illness-related stigma among adolescent student, but might predict higher levels of stigma, particularly among university students. This suggests that educational interventions focusing solely on knowledge dissemination, without addressing emotional attitudes and social norms, may not only fail to effectively reduce stigma but could potentially produce counterproductive effects. Future anti-stigma strategies should adopt more integrated approaches and pay special attention to the psychosocial characteristics of students at different developmental stages.

## 1 Introduction

Today, adolescent mental health presents a critical public health challenge globally (1). Anxiety disorders affect 4.1% of adolescents aged 10–14 and 5.3% of those aged 15–19, while depression affects 1.3% and 3.4% respectively (2). However, despite these numbers, up to 75% of adolescents worldwide fail to receive the professional help they need (3). The primary barrier to receiving care is mental illness-related stigma, which encompasses both personal shame associated with having a disorder, and societal negative attitudes (e.g., stereotypes, prejudice) and discriminatory behaviors (4). The impact of stigma often surpasses that of the illnesses themselves (5), which is especially true in adolescence when one is in a critical period for identity formation (6, 7). Stigma severely inhibits help-seeking intentions leading to concealment and avoidance behaviors (4, 8). Furthermore, it initiates a vicious cycle of the “labeling effect,” exacerbating public cognitive biases and structural discrimination (5, 9), thereby generating a cascade of negative consequences for adolescents’ social functioning and long-term prognosis (3, 10).

In the Chinese context, mental illness-related stigma is particularly pronounced due to deep-seated cultural beliefs that associate mental health problems with personal weakness, family shame, or even moral failing, heavily influenced by collectivistic values that prioritize social harmony and “saving face” (11). Consequently, the internalization of public stigma (i.e., self-stigma) among Chinese adolescents is a major driver of low help-seeking rates (12, 13). Concurrently, mental health knowledge, often subsumed under the broader concept of mental health literacy (MHL), remains inadequate (14). Despite recent governmental efforts to promote mental health education in schools, the content often lacks specificity and fails to effectively target the core components of stigma, such as challenging the dangerousness stereotype (12, 14). This situation mirrors that of other Asian societies with similar cultural backgrounds. For example, a survey in Tokyo revealed that although personal stigma among adolescents is lower than that of their parents, it remains prevalent, while family exposure experience can effectively reduce such stigma (15). In South Korea, face culture, self-stigma, and a lack of mental health knowledge collectively weaken help-seeking intentions and exacerbate the interaction of stigma (16). The high levels of stigma, significant treatment gaps, and fear of labeling intensified by familiarity and academic pressure observed among adolescents in both Japan and South Korea reflect regional commonalities and underscore the urgent need for culturally adapted intervention measures.

To better understand the landscape in which stigma and knowledge interact, it is important to consider the primary sources of mental health information for Chinese adolescents and the nature of existing educational attempts. The school environment serves as the main formal channel for mental health knowledge dissemination, primarily through designated curricula, occasional lectures, and school-wide publicity campaigns (17). However, as noted, the content is often generalized. Concurrently, adolescents increasingly access information through digital media, including social platforms (e.g., Weibo, Douyin, Bilibili), online health forums, and entertainment media, which present unfiltered and sometimes sensationalized or stigmatizing portrayals of mental illness (18). Information from family and peers, while potentially supportive, is often colored by prevailing cultural stigma and misconceptions (19). Current attempts to enhance mental health knowledge in China largely follow a top-down, curriculum-based approach focused on psychoeducation regarding common disorders and stress management (20). While mandatory mental health education classes have been implemented in many schools, their frequency, depth, and quality vary considerably. Moreover, these efforts rarely employ evidence-based anti-stigma strategies (e.g., structured contact with individuals in recovery, targeted cognitive restructuring) and infrequently address the specific developmental needs of students at different educational stages (18, 21). This fragmented information environment, coupled with educational interventions that may not directly counter stigma, creates a complex context for understanding how knowledge and stigma influence each other over time.

Conventional theory posits that increasing knowledge about mental illness can reduce mental illness-related stigma, a premise known as the “knowledge diminishes prejudice” model (22). Theoretical frameworks, such as the Attribution Model, suggest that providing biogenetic explanations for mental illness can reduce blame but might inadvertently increase perceptions of persistence and dangerousness, thereby complicating the relationship between knowledge and stigma (23). Some studies support this view, indicating that improved mental health literacy contributes to less stigmatizing attitudes and promotes help-seeking behaviors (24, 25). Conversely, other research findings suggest that greater knowledge does not necessarily lead to a reduction in stigma. For instance, a study involving international students found that better knowledge of schizophrenia was associated with higher levels of perceived stigma (26). Similarly, within the mental health domain, a study found that certain types of knowledge, particularly a detailed understanding of symptoms and prognosis, can sometimes heighten the public’s perception of individuals with mental illness as unpredictable or dangerous, thereby strengthening rather than weakening stigmatizing attitudes (27). While a deeper understanding of an illness may lead to a clearer perception of its severity and risks, such heightened awareness could instead be adversely fostering and reinforcing fear, a significant root cause of stigma and discrimination (28). In contrast, knowledge that aims to dissociate mental illness with “dangerousness” is more conducive to dispelling misconceptions, thereby reducing stigma (26). Furthermore, the Enhanced Contact Model (ECM) proposed by Ran et al. in 2018 (29) demonstrated that this approach was more effective than pure knowledge-based education in reducing affiliate stigma among family members of individuals with schizophrenia (30). This highlights that the efficacy of knowledge-based interventions is not guaranteed and may be theoretically moderated by the nature of the knowledge and the delivery method. Given this complexity, schools, as the central setting for identification and intervention, must account for the developmental differences across educational stages (31). Notably, junior high school students (aged 12–15) are highly influenced by peers and authority figures, leading to a tendency to understand mental illness in stigmatizing, labeled terms (32). Moreover, although senior high school students (aged 15–18) begin to adopt more abstract thinking, academic pressure may lead students to negatively associate mental illness with personal failure (33). Lastly, while university students (aged 18–22) possess mature cognitive abilities, identity exploration and social comparison during this period can significantly intensify the internalization of stigma (34). The current education system has a narrow focus, predominantly emphasizing knowledge transmission, and university level support systems are often insufficient, all which may be hindering the effective translation of knowledge into positive attitudes (3).

Evidence from cross-sectional studies confirms the association between mental illness-related stigma and mental health knowledge levels, but the direction of causality remains unclear. Moreover, findings across existing studies are inconsistent, showing a need to clarify the causal relationship between these two variables, which would be crucial for designing effective anti-stigma interventions. Additionally, this relationship may be significantly moderated by adolescent developmental stage; however, a systematic examination of this potential moderating effect is lacking in the literature. This gap has resulted in a lack of a robust evidence for designing interventions tailored to different age groups. To address these research gaps, this study employed a two-wave longitudinal survey design and utilized a cross-lagged panel model. The primary objectives of this study are: 1) To determine the longitudinal, bidirectional causal relationships between mental illness-related stigma and mental health knowledge in a sample of Chinese adolescents; and 2) To examine whether these cross-lagged paths are moderated by educational stage (i.e., junior high school, senior high school, and university). Our central research questions are: a) Does prior stigma predict subsequent knowledge levels, or does prior knowledge predict subsequent stigma levels? b) Does the strength or direction of these predictive relationships differ significantly across the three educational stages? These findings will provide key scientific evidence for developing highly efficient and precise mental health promotion and anti-stigma interventions “tailor-made” for adolescents of different ages. This evidence will directly inform school-based mental health education policies and public health strategies.

## 2 Methods

### 2.1 Participants and sampling procedure

This study employed a longitudinal design with data collected from the same adolescent cohort at two time points: the baseline survey (T1) was conducted from December 2022 to March 2023, and the follow-up survey (T2) was completed between December 2023 and April 2024. A multi-stage, stratified, cluster sampling method was employed to enhance the representativeness of the sample. First, five cities in Sichuan Province (Chengdu, Ya’an, Mianyang, Guangyuan, and Dazhou) were purposively selected to capture geographical (e.g., central, west, north, east) and socioeconomic diversity. Second, within each city, a convenience sampling method was used to recruit schools that were willing and available to participate, ensuring coverage of the three target educational stages (junior high school, senior high school, and university). Ultimately, 28 schools were included. The study initially involved 7345 students from these schools. Clear inclusion and exclusion criteria were established. Inclusion criteria were: 1) aged 12–24 years; 2) voluntary participation with agreement to follow-up; 3) normal intelligence with the ability to complete questionnaires appropriately; and 4) ability to conscientiously complete the questionnaire without omissions. Exclusion criteria included: 1) diagnosis of severe physical diseases (e.g., cardiovascular diseases, cerebrovascular diseases, malignant tumors) that could significantly impair daily functioning or cognitive capacity, or that were likely to introduce confounding severe stress unrelated to the study’s focus on mental health; 2) presence of severe cognitive impairment or intellectual disability (typically corresponding to an IQ <70) that would preclude valid comprehension of the survey items; 3) diagnosis of other serious mental disorders (e.g., psychotic disorders, severe bipolar disorder) as these conditions could fundamentally alter the perception and reporting of stigma and knowledge, and their inclusion would require a distinct analytical framework; and 4) attrition during the study or incomplete data collection. The identification of participants meeting exclusion criteria 1–3 was primarily based on: a) self-report or parent/guardian report via a brief health screening checklist administered prior to the main survey; and b) available school health records for students who reported or were known to have significant medical or psychological conditions. Participants flagged by either method were respectfully excluded to maintain sample homogeneity and ensure that the measured constructs (stigma and knowledge) reflected general adolescent perceptions rather than the direct, severe sequelae of major health conditions.

Prior to the commencement of the study, ethical approval was obtained from the Biomedical Research Ethics Committee of West China Hospital of Sichuan University (Approval No: 2022-1790).Written informed consent was obtained from all participants or their legal guardians.

After screening, 5579 participants were included in the final valid sample, comprising 1695 junior high school students, 3120 senior high school students, and 764 university students; a total of 1766 participants were excluded due to loss to follow-up or failure to meet the study criteria.

### 2.2 Implementation and measures

To ensure data quality and minimize biases during data collection, a standardized protocol was implemented. First, the research team conducted systematic training for teachers from the participating schools. The training, delivered by professional research assistants, covered scale administration guidelines, precautions, and solutions to common issues, aiming to ensure that all teachers accurately understood the assessment procedures and could standardize the distribution and on-site supervision of questionnaires. Specifically, teachers were instructed to use a neutral tone, avoid guiding students’ responses, and ensure a quiet, independent testing environment to minimize social desirability bias and interferences.

During the data collection phase, the trained teachers uniformly organized the process in classroom settings. They distributed the questionnaires to each student and instructed them to complete the surveys honestly and as required. Teachers provided continuous supervision throughout the process, thereby effectively ensuring the authenticity and completeness of the data collected. Furthermore, participants were assured of the confidentiality and anonymity of their responses to encourage honest answering.

#### 2.2.1 Demographic data

A self-administered demographic questionnaire was used to collect students’ basic information, including name, age, sex, ethnicity, domicile, accommodation type, relationship status, family living arrangement, objective monthly income, subjective economic status, only child status, parental marital status, parental education and parenting styles.

#### 2.2.2 Mental illness-related stigma

The level of mental illness-related stigma was assessed using the Chinese version of the Perceived Devaluation-Discrimination Scale (PDD) (35). The PDD comprises two subscales: “Perceived Devaluation” and “Perceived Discrimination.” The former reflects an individual’s expectation of negative views held by others toward people with mental illness (e.g., being seen as dangerous or untrustworthy), while the latter refers to the anticipation of unfair treatment (e.g., being distanced or denied opportunities). The scale consists of 12 items rated on a 4-point scale (1 = strongly agree, 4 = strongly disagree). Items 7 to 12 are reverse-scored. The total score is the sum of all items, ranging from 12 to 48, with higher scores indicating a greater perceived level of stigma. The Chinese version of the PDD has demonstrated good reliability and validity in previous studies (36). In the current study, the scale showed good internal consistency, with Cronbach’s α coefficients of 0.776 at T1 and 0.759 at T2.

#### 2.2.3 Mental health knowledge

The level of mental health knowledge was assessed using the Chinese version of the Mental Health Knowledge Schedule (MAKS) (37). This 12-item scale is divided into two dimensions: the first six items measure core knowledge of mental health, and the remaining six items assess the ability to identify common mental illnesses. Responses are recorded on a 5-point Likert scale ranging from 1 (strongly disagree) to 5 (strongly agree). Items 6, 8, and 12 are reverse-scored. The total score is the sum of all items, ranging from 12 to 60, with higher scores indicating a greater level of mental health knowledge. The scale has demonstrated good reliability and validity in Chinese adolescent populations (38). In the present sample, the internal consistency was acceptable, with Cronbach’s α coefficients of 0.829 at T1 and 0.811 at T2.

### 2.3 Statistical analysis

Data analysis was performed using R software. The raw data underwent rigorous cleaning and preprocessing to ensure data quality. Participants were then stratified into three groups according to their educational stage: junior high school, senior high school, and university. The Kruskal-Wallis rank sum test and Pearson’s chi-squared test were employed to compare differences in sociodemographic characteristics, MAKS scores, and PDD scores across these groups at both T1 and T2 time points, aiming to elucidate the basic characteristics and distribution patterns of psychological variables among students at different stages. Furthermore, correlation matrices between MAKS and PDD scores for each group at T1 and T2 were visualized using the corrplot package to identify associated factors. Finally, to examine the causal relationship between the two variables while accounting for potential confounding effects, cross-lagged panel models (CLPM) were constructed with the lavaan package. These models incorporated MAKS and PDD scores from both T1 and T2 as primary variables. Crucially, based on the significant between-group differences observed in our descriptive analysis, a set of key demographic covariates that could influence both knowledge and stigma were included in all models. These variables were entered into the models to statistically control for their effects, allowing us to estimate the cross-lagged paths between MAKS and PDD net of these potential confounders. A two-sided P value of less than 0.05 was considered statistically significant for all tests.

## 3 Results

### 3.1 Descriptive statistical analysis

**Table 1** details the demographic characteristics, PDD, and MAKS scores across the three educational stages. Demographic analyses revealed significant between-group differences (p < 0.05) for all variables. As expected, mean age increased with educational stage (13.99 ± 0.84, 16.67 ± 0.71, 19.52 ± 0.71 years; p < 0.001). Notable trends included an increasing proportion of female students (54.57%, 67.28%, 68.85%; p < 0.001) and students in romantic relationships (1.24%, 2.12%, 20.81%; p < 0.001) with higher educational stages. The proportion of students self-identifying with a “below average” subjective economic status also increased (21.06%, 24.46%, 37.17%; p < 0.001). University students had a significantly higher proportion of ethnic minorities (7.07% vs. 1.95% and 3.56%; p < 0.001), while senior high students had the highest proportion from rural domiciles (86.57%; p < 0.001). Junior high students showed the highest rates of only-child status (31.33%; p < 0.001) and authoritative parenting (68.32%; p < 0.001). Parental education was generally low, particularly among university students’ parents (fathers: 90.97%; mothers: 95.03% with high school education or below; p < 0.001). Significant differences were also observed in accommodation type, family living arrangements, objective monthly income, and parental marital status (all p < 0.05).

**Table 1:** A comparison of demographic characteristics, mental illness-related stigma, and mental health literacy in adolescents across junior high, senior high, and university.

Characteristic	Junior high school	Senior high school	University	p-value2
	N = 1,695	N = 3,120	N = 764	
Age	13.99 (0.84)	16.67 (0.71)	19.52 (0.71)	<0.001
Gender				<0.001
Male	770(45.43%)	1,021 (32.72%)	238 (31.15%)	
Female	925 (54.57%)	2,099 (67.28%)	526 (68.85%)	
Ethnicity				<0.001
Han	1,662 (98.05%)	3,009 (96.44%)	710 (92.93%)	
Minorities	33 (1.95%)	111 (3.56%)	54 (7.07%)	
Domicile				<0.001
Rural	1,319 (77.82%)	2,701 (86.57%)	599 (78.40%)	
Urban	376 (22.18%)	419 (13.43%)	165 (21.60%)	
Accommodation type				<0.001
Day School	165 (9.73%)	260 (8.33%)	5 (0.65%)	
Boarding School	1,530 (90.27%)	2,860 (91.67%)	759 (99.35%)	
Relationship Status				<0.001
Single	1,674 (98.76%)	3,054 (97.88%)	605 (79.19%)	
In a relationship	21 (1.24%)	66 (2.12%)	159 (20.81%)	
Family Living Arrangement				0.025
Parents	1,187 (70.03%)	2,068 (66.28%)	518 (67.80%)	
Grandparents	282 (16.64%)	550 (17.63%)	117 (15.31%)	
Other	226 (13.33%)	502 (16.09%)	129 (16.88%)	
Objective Monthly Income				<0.001
≤4999	862 (50.86%)	1,714 (54.94%)	487 (63.74%)	
5000-19999	697 (41.12%)	1,243 (39.84%)	254 (33.25%)	
>20000	136 (8.02%)	163 (5.22%)	23 (3.01%)	
Subjective Economic Status				<0.001
Above Average	64 (3.78%)	30 (0.96%)	12 (1.57%)	
Average	1,274 (75.16%)	2,327 (74.58%)	468 (61.26%)	
Below Average	357 (21.06%)	763 (24.46%)	284 (37.17%)	
Only child?				<0.001
Yes	531 (31.33%)	600 (19.23%)	164 (21.47%)	
No	1,164 (68.67%)	2,520 (80.77%)	600 (78.53%)	
Parental Marital Status				0.042
Single	25 (1.47%)	42 (1.35%)	16 (2.09%)	
Married	1,408 (83.07%)	2,518 (80.71%)	641 (83.90%)	
Divorced	141 (8.32%)	290 (9.29%)	65 (8.51%)	
Remarried	95 (5.60%)	228 (7.31%)	34 (4.45%)	
Other	26 (1.53%)	42 (1.35%)	8 (1.05%)	
Father’s Education				<0.001
Primary School or Below				
Junior High School				
High School or Vocational School				
Associate Degree or Above	264 (15.58%)	194 (6.22%)	69 (9.03%)	
Mother’s Education				<0.001
Primary School or Below	440 (25.96%)	1,123 (35.99%)	326 (42.67%)	
Junior High School	658 (38.82%)	1,357 (43.49%)	299 (39.14%)	
High School or Vocational School	342 (20.18%)	485 (15.54%)	101 (13.22%)	
Associate Degree or Above	255 (15.04%)	155 (4.97%)	38 (4.97%)	
Parenting Styles				<0.001
Authoritative	1,158 (68.32%)	1,769 (56.70%)	434 (56.81%)	
Authoritarian	343 (20.24%)	761 (24.39%)	163 (21.34%)	
Neglectful	39 (2.30%)	189 (6.06%)	54 (7.07%)	
Permissive	155 (9.14%)	401 (12.85%)	113 (14.79%)	
T1-MAKS	27.27 (8.74)	27.84 (8.58)	26.97 (7.68)	0.006
T2-MAKS	41.31 (4.95)	40.98 (4.87)	42.41 (4.89)	<0.001
T1-PDD	30.14 (7.76)	29.69 (7.21)	29.98 (6.15)	0.042
T2-PDD	29.30 (3.35)	29.29 (3.06)	29.69 (3.15)	0.002

^1^n (%); Mean (SD); 2Kruskal-Wallis rank sum test; Pearson’s Chi-squared test.

Regarding the core study variables, significant between-group differences were found at both time points (all p < 0.05). For mental health knowledge (MAKS), scores improved significantly from T1 to T2 across all groups. At T1, senior high students scored highest (27.84 ± 8.58, p = 0.006), while at T2, university students scored highest (42.41 ± 4.89, p < 0.001). For stigma (PDD), scores decreased from T1 to T2 in all groups. Junior high students reported the highest stigma levels at T1 (30.14 ± 7.76, p = 0.042), whereas university students showed the highest levels at T2 (29.69 ± 3.15, p = 0.002).

### 3.2 Correlation analysis

**Figure 1:**
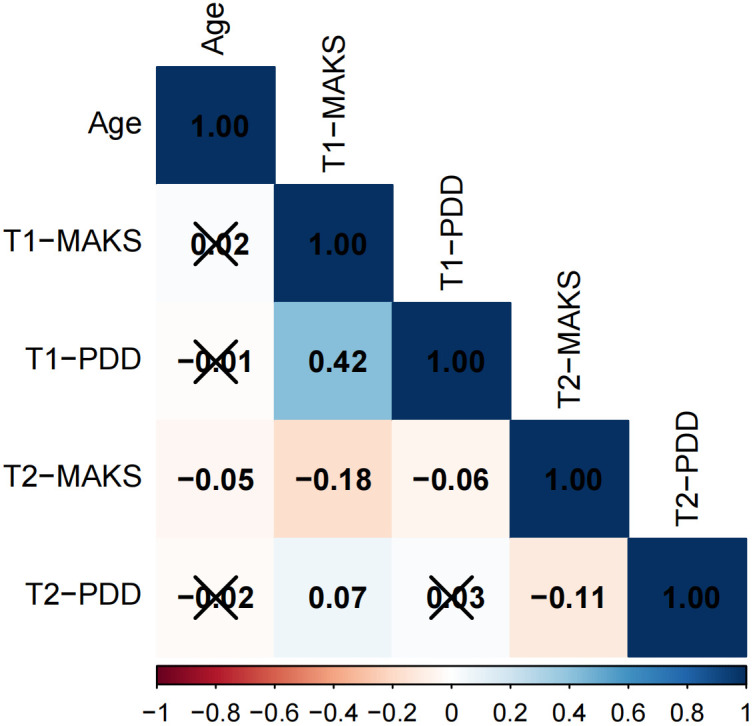
Correlation analysis of junior high school students.

**Figure 2:**
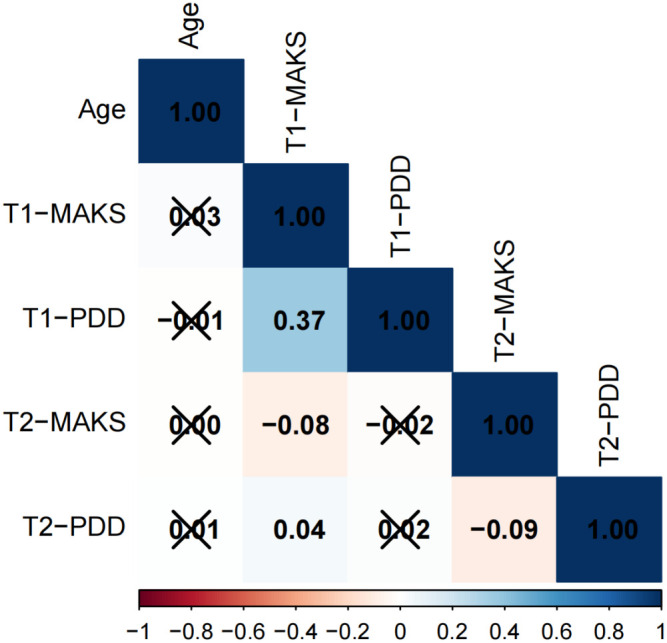
Correlation analysis of senior high school students.

**Figure 3:**
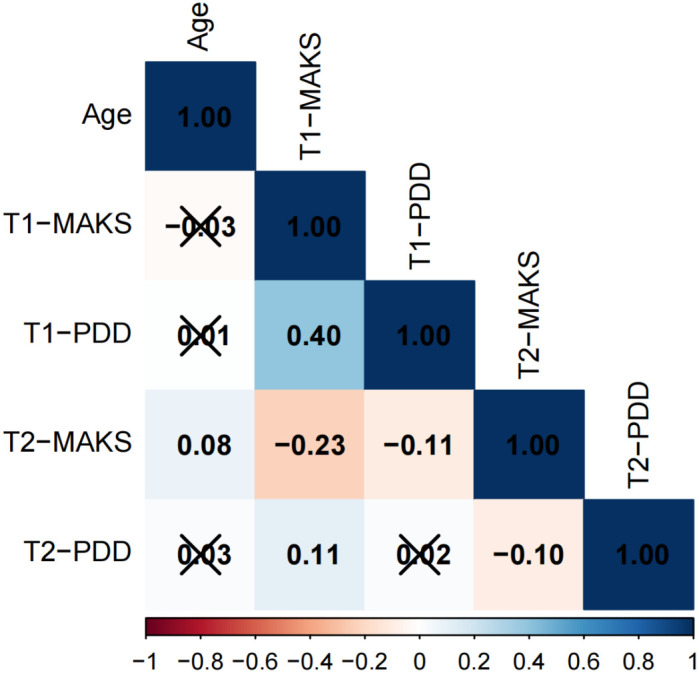
Correlation analysis of university students.

The results of the correlation analysis are presented in **Figures 1**–**3**. The data is presented in the form of correlation coefficients. Among adolescents across different educational stages, T1-PDD showed significant positive correlations with T1-MAKS (junior high: r = 0.42; senior high: r = 0.37; university: r = 0.40). T2-MAKS was significantly negatively correlated with T1-MAKS (junior high: r = –0.18; senior high: r = –0.08; university: r = –0.23), while T2-PDD was significantly positively correlated with T1-MAKS (junior high: r = 0.07; senior high: r = 0.04; university: r = 0.11). In addition, T2-PDD was significantly negatively correlated with T2-MAKS across all groups (junior high: r = –0.11; senior high: r = –0.09; university: r = –0.10). In the junior high school group, T2-MAKS was significantly negatively correlated with both age and T1-PDD (r = –0.05 and –0.06, respectively). In the university group, T2-MAKS was significantly positively correlated with age (r = 0.11), but significantly negatively correlated with T1-PDD (r = –0.10).

### 3.3 Cross-lagged panel modeling analysis

**Figure 4:**
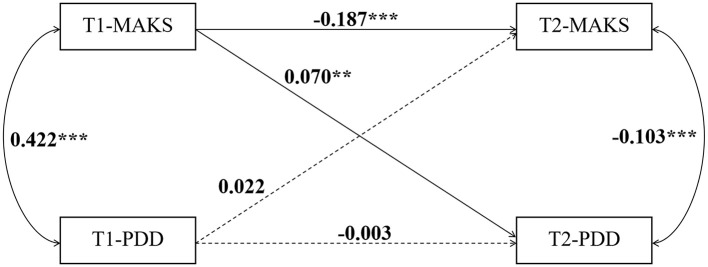
The causal relationship between MAKS and PDD in junior high school students. **p < 0.01, ***p < 0.001.

**Figure 5:**
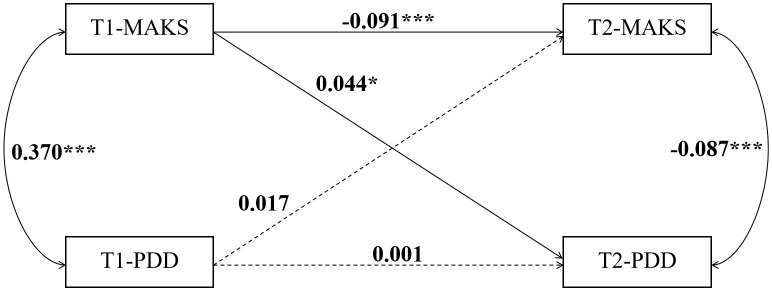
The causal relationship between MAKS and PDD in senior high school students. *p < 0.05, ***p < 0.001.

**Figure 6:**
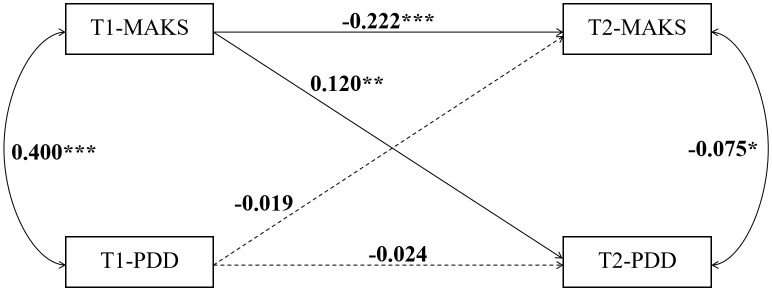
The causal relationship between MAKS and PDD in university students. *p < 0.05, **p < 0.01, and ***p < 0.001.

To clarify the causal relationship between MAKS and PDD, we constructed cross-lagged panel models for the three groups—junior high, senior high, and university students (**Figures 4**–**6**). The data is presented using standardized coefficients. The models demonstrated acceptable goodness-of-fit, and the results indicated that, across all three groups, T1 MAKS significantly and positively predicted T2 PDD (junior high: β = 0.070, p < 0.01; senior high: β = 0.044, p < 0.05; university: β = 0.120, p < 0.01). Meanwhile, T1 MAKS also significantly and negatively predicted T2 MAKS (junior high: β = –0.187, p < 0.001; senior high: β = –0.091, p < 0.001; university: β = –0.222, p < 0.001). Furthermore, at the T1 time point, MAKS scores were significantly positively correlated with PDD scores across all three educational stage groups (junior high: β = 0.422, p < 0.001; senior high: β = 0.370, p < 0.001; university: β = 0.400, p < 0.001). In contrast, at the T2 time point, MAKS scores showed significant negative correlations with concurrent PDD scores (junior high: β = -0.103, p < 0.001; senior high: β = -0.087, p < 0.001; university: β = -0.075, p < 0.05).

## 4 Discussion

This study investigated the bidirectional causal relationship between mental illness knowledge and mental illness-related stigma among Chinese adolescents in Sichuan Province across different educational stages (junior high, senior high, and university) through a two-wave longitudinal survey utilizing a cross-lagged panel model. The key finding noted that a higher level of mental health knowledge at baseline (T1) consistently predicted a higher level of mental illness-related stigma at follow-up (T2) across all three stages. This finding directly challenges the traditional “knowledge diminishes prejudice” hypothesis (24, 25)and provides supporting evidence for the superiority of the Enhanced Contact Model (ECM) (29).

The results revealed that a higher MAKS score at T1 significantly predicted a higher PDD score at T2 among adolescents across junior high, senior high, and university stages. This suggests that a higher initial level of mental health knowledge may be exacerbating subsequent mental illness-related stigma through complex mechanisms. This finding challenges the results demonstrated in a majority of studies which report a negative correlation between MAKS and PDD (35, 39–41), and from intervention trials that found no significant association (42)—42)IN interpreted as evidence that knowledge must be combined with behavioral strategies to effectively improve attitudes. Conversely, the findings of the present study align with those of several other reports (43, 44). This inconsistency may stem from differences in study populations, assessment tools, and cultural contexts, which collectively underscore the uniqueness and complexity of the “knowledge-attitude” relationship within adolescent populations and different cultural backgrounds. This positive relationship may be explained by several mechanisms. First, a “cognitive awakening effect” may be at play, whereby increased mental health knowledge heightens adolescents’ sensitivity to discriminatory phenomena in reality, which is consequently reflected in higher PDD scores (42). From a social psychological perspective, this aligns with and extends the concepts of “system justification theory” and “social identity theory” (45). As adolescents’ cognitive awareness of mental illness and its associated stigma becomes more sophisticated, they may become more acutely aware of the existing negative stereotypes and discriminatory norms within their social environment (e.g., schools, society) (46). This heightened awareness, rather than reducing personal endorsement of stigma, might first manifest as an increased perception of how pervasive and legitimate these stigmatizing attitudes are in the public sphere (i.e., perceived devaluation and discrimination), which is precisely what the PDD measures (47). This is particularly salient during adolescence, a period intensely focused on social belonging and peer perception (46, 48). Second, there might be an inherent content overlap in the measurement tools themselves—items in the MAKS pertaining to societal treatment (e.g., “people with mental illness are often treated unfairly”) intersect with the perceptions of discrimination assessed by the PDD, thereby potentially amplifying the covariance between the two constructs (39, 42). Furthermore, this finding can be interpreted through the lens of “attribution theory.” If the acquired knowledge overemphasizes biogenetic explanations without contextualizing recovery and personal agency, it might inadvertently foster perceptions of mental illness as chronic, fundamental, and dangerous differences,yng components of stigma (49, 50). Similarly, “labeling theory” suggests that learning the diagnostic labels and symptoms can solidify the “us vs. them” boundary, making the category of “mentally ill” more salient and facilitating the application of negative stereotypes, especially in a formative developmental stage where identity is fluid and vulnerable (51). Furthermore, as adolescents are in a critical period of identity formation, an increase in knowledge might initially sharpen their awareness of the risk of social exclusion, rather than leading to an immediate positive shift in personal attitudes (52).

Two important methodological considerations must be integrated into the interpretation of our core findings. First, we observed a substantial mean increase in MAKS scores from T1 to T2 across all groups. While this could reflect true gains due to maturation, cumulative education, or increased societal awareness, the magnitude of change warrants consideration of alternative explanations. These include testing effects or reactivity (where T1 assessment sensitized participants to seek information) (53), reduction in social desirability bias over time (54), or the measurement properties of the MAKS itself (55). Second, it is crucial to distinguish between two distinct longitudinal patterns. Our primary findings.nal T1 MAKS positively predicts T2 PDDdictslyal the potential “backfire” effect discussed above. Separately, we observed that lower T1 MAKS scores predicted higher T2 MAKS scores (i.e., a negative autoregressive path). This pattern likely reflects statistical phenomena such as regression to the mean (56) or a ceiling effect (57) for high scorers at T1, indicating that adolescents with initially lower knowledge showed greater relative gain over time. It is important to note that this pattern of knowledge change is conceptually and statistically independent from the core relationship between knowledge and stigma.

Furthermore, this study also found that the MAKS score at T1 negatively predicted the MAKS score at T2, indicating that the acquisition of knowledge may not be a simple linear process but could involve cognitive restructuring or information conflict (58). This phenomenon could be explained by natural knowledge decay (59), characteristics of the assessment tool (60), or a psychological avoidance mechanism (61). Specifically, if an initial exposure to knowledge is accompanied by an increase in mental illness-related stigma, it may induce psychological discomfort or reactance, leading to subsequent intentional avoidance of related information rather than active assimilation and integration.

The causal relationship path from “mental health knowledge → mental illness-related stigma” was consistent across all developmental stages, indicating the robustness of this pathway among the adolescent population, transcending developmental stages. However, the effect size of the path differed across the three groups (junior high, senior high, and university), with the strongest effect observed in university students. The prominent effect may stem from stage-specific psychosocial challenges. As university students are in a critical period of identity exploration, complex social comparisons, and risk for future employment discrimination (62–64), they may have a heightened sensitivity to negative information associated with mental illness.

In summary, by integrating a developmental psychology perspective, this study demonstrates that the causal relationship between mental health knowledge and mental illness-related stigma persists across different adolescent stages, with its effect strength varying with age. The scientific contributions of this research are threefold. First, it provides crucial longitudinal evidence that challenges the universal applicability of the “knowledge diminishes prejudice” model in adolescent mental health promotion, highlighting a potential iatrogenic effect of knowledge-only approaches. Second, methodologically, it introduces a valuable developmental perspective by demonstrating how this causal relationship is moderated by educational stage, thereby moving beyond a one-size-fits-all understanding. Third, it offers a theoretically grounded explanation by integrating social psychology frameworks (e.g., social identity, attribution theory) to elucidate the underlying mechanisms, thereby enriching the conceptual discourse on the knowledge-stigma relationship. This finding raises a critical caution for current school-based mental health education practices: interventions that focus solely on knowledge dissemination while neglecting the careful curation of content and active addressing of stigma, as it may counterproductively trigger defensive cognitions. For instance, an increase in knowledge may risk heightening an individual’s vigilance against “being labeled” and “social exclusion,” rather than directly improving attitudes (65), especially within China’s collectivist cultural context. Therefore, our findings suggest a need to critically re-evaluate interventions that rely exclusively on knowledge transmission. The longitudinal association we observed—where greater knowledge predicted increased stigma—highlights a potential unintended consequence that should be mitigated. This evidence conceptually supports the development and evaluation of more comprehensive intervention frameworks that move beyond psychoeducation alone. Such frameworks, which may include components like structured contact with individuals with lived experience (as in the Enhanced Contact Model, ECM), skills training, and activities designed to directly counter stereotypes, aim to address the affective and behavioral roots of stigma, not just its cognitive components. Concurrently, interventions must be “tailored to developmental stages,” with particular attention paid to university students, who, although cognitively mature, may be especially vulnerable to the internalization of stigma due to pressures surrounding identity exploration and social competition (66).

## 5 Limitation

This study has several limitations: 1) First and foremost, a critical methodological limitation pertains to the potential conceptual overlap between our two primary measures. MAKS includes items that assess perceptions of how society treats people with mental illness (e.g., “people with mental illness are often treated unfairly”), which conceptually intersects with the construct of perceived public stigma measured by the PDD. This overlap may have artificially inflated the concurrent correlations observed between MAKS and PDD scores and could potentially bias the estimation of the cross-lagged paths, leading to an overestimation of their longitudinal relationship. Future studies should employ knowledge measures with minimal attitudinal content or conduct analyses using purified subscales to better disentangle the unique contributions of knowledge and stigma perceptions. 2) First, although it employed a longitudinal design, it remains an observational study, and thus the influence of unmeasured confounding variables cannot be entirely ruled out; 3) while the sample possessed internal diversity, all participants were recruited from Sichuan Province; therefore, caution is warranted when generalizing the findings to adolescents from other cultural and socioeconomic backgrounds across China; 4) the reliance on self-report measures introduces the potential for social desirability bias; 5) the approximate one-year interval between the two measurement waves may have captured a specific change trajectory; shorter or longer intervals might yield different patterns of stability, necessitating future studies with multiple time points to elucidate the dynamic processes involved. 6) the sample was restricted to students currently enrolled in formal education, thereby not encompassing individuals who had dropped out of school or were not attending any educational institution. Notably, the absence of participants who did not pursue university education is a significant constraint, as this population may in fact represent a group with greater need for mental health research and intervention.

## 6 Conclusion

This study collected longitudinal data at two time points from the same cohort of adolescents in Sichuan Province, China, to investigate the causal relationship between mental illness knowledge and mental illness-related stigma among adolescents at different educational stages. The main findings are as follows: 1) Among contemporary Chinese adolescents, an increase in mental health knowledge may unexpectedly lead to an exacerbation of stigma, an effect that is particularly pronounced at the university stage; 2) An increase in adolescents’ mental health knowledge may predict a decrease in mental health knowledge one year later. These findings pose a significant challenge to the traditional “knowledge diminishes prejudice” intervention paradigm and reveal a potential for psychological avoidance or cognitive conflict mechanisms to arise during knowledge internalization. Accordingly, future research should establish long-term tracking at multiple time points to capture the dynamic evolution of this causal relationship. Further exploration of the underlying mechanisms and the development of precise interventions, such as the Enhanced Contact Model (ECM), are essential steps to promoting adolescent mental health and alleviating stigma.

## Data Availability

The raw data supporting the conclusions of this article will be made available by the authors, without undue reservation.
